# From Default Mode Network to the Basal Configuration: Sex Differences in the Resting-State Brain Connectivity as a Function of Age and Their Clinical Correlates

**DOI:** 10.3389/fpsyt.2018.00365

**Published:** 2018-08-13

**Authors:** Sean D. Conrin, Liang Zhan, Zachery D. Morrissey, Mengqi Xing, Angus Forbes, Pauline Maki, Mohammed R. Milad, Olusola Ajilore, Scott A. Langenecker, Alex D. Leow

**Affiliations:** ^1^Department of Psychiatry, University of Illinois at Chicago, Chicago, IL, United States; ^2^Department of Electrical and Computer Engineering, University of Pittsburgh, Pittsburgh, PA, United States; ^3^Department of Engineering and Technology, University of Wisconsin-Stout, Menomonie, WI, United States; ^4^Department of Bioengineering, University of Illinois at Chicago, Chicago, IL, United States; ^5^Department of Computational Media, University of California, Santa Cruz, Santa Cruz, CA, United States

**Keywords:** brain connectivity, sex differences, human connectome project, modularity, resting-state fMRI, default-mode network, community structure

## Abstract

Connectomics is a framework that models brain structure and function interconnectivity as a network, rather than narrowly focusing on select regions-of-interest. MRI-derived connectomes can be structural, usually based on diffusion-weighted MR imaging, or functional, usually formed by examining fMRI blood-oxygen-level-dependent (BOLD) signal correlations. Recently, we developed a novel method for assessing the hierarchical modularity of functional brain networks—the probability associated community estimation (PACE). PACE uniquely permits a dual formulation, thus yielding equivalent connectome modular structure regardless of whether positive or negative edges are considered. This method was rigorously validated using the 1,000 functional connectomes project data set (F1000, RRID:SCR_005361) (1) and the Human Connectome Project (HCP, RRID:SCR_006942) (2, 3) and we reported novel sex differences in resting-state connectivity not previously reported. (4) This study further examines sex differences in regard to hierarchical modularity as a function of age and clinical correlates, with findings supporting a basal configuration framework as a more nuanced and dynamic way of conceptualizing the resting-state connectome that is modulated by both age and sex. Our results showed that differences in connectivity between men and women in the 22–25 age range were not significantly different. However, these same non-significant differences attained significance in both the 26–30 age group (*p* = 0.003) and the 31–35 age group (*p* < 0.001). At the most global level, areas of diverging sex difference include parts of the prefrontal cortex and the temporal lobe, amygdala, hippocampus, inferior parietal lobule, posterior cingulate, and precuneus. Further, we identified statistically different self-reported summary scores of inattention, hyperactivity, and anxiety problems between men and women. These self-reports additionally divergently interact with age and the *basal configuration* between sexes.

## Introduction

In efforts to better understand the human connectome, various approaches have been used to identify and measure the modularity of brain connectivity. In these efforts the brain is generally divided into a collection of communities or “modules.” Frequently, these modules can be sub-divided into submodules, which then demonstrate hierarchical modularity and *near decomposability* (the autonomy of modules from one another) (5). Modules and sub-modules are comprised of a series of nodes with tight interconnectivity, whereas nodes of different modules have lesser connectivity. The connections between nodes are referred to as edges and can be either positive or negative in fMRI Connectomics. A positive edge indicates that the activity in one node is positively correlated with that in the connected node, whereas a negative edge indicates the presence of an inverse relationship between the two (6). When these concepts are applied to fMRI-derived networks, network organization identifies functionally related or “coupled” regions.

The complexity and volume of data associated with large networks is enormous and thus much work has been done to develop algorithms that better characterize and measure modularity (7). In the popular approach of maximizing the Q modularity metric, which is an NP-hard problem, typically the speed of computation comes with a compromise (8). For example, the very efficient fast unfolding method does not guarantee global optimization, and yields a varying number of communities with each run (9). Another area of variations is how positive and negative edges are accounted for. More frequently, the focus of computation is based on the recognition and measure of positive edges as this is fitting for many types of networks (10, 11). However, in measuring brain connectivity through fMRI networks, which usually focuses on fMRI blood-oxygen-level-dependent **(BOLD)** signal correlations, positive and negative edges co-exist.

Most published studies have employed variable approaches of ignoring, thresholding, binarizing, or arbitrary down-weighting to account for these negative edges (12–14). Although quite different in their approaches, the common similarity is that the data involving negative edges is in some degree heuristically accounted for.

To better address negative edges, we recently developed and published a novel method for assessing the modularity of functional brain networks—the probability associated community estimation (PACE) (4). Most importantly, PACE permits a dual formulation, thus yielding equivalent connectome modular structure regardless of whether one considers positive or negative edges, by exploiting how frequent BOLD signal correlation between two regions is positive vs. negative (the edge “positivity” and “negativity”). This method was rigorously validated using resting-state fMRI data from the 1,000 functional connectomes or F1000 project data set (F1000, RRID:SCR_005361) (1) and the Human Connectome Project (HCP, RRID:SCR_006942) (2, 3) and we demonstrated that negative correlations alone are sufficient in understanding resting-state connectome modularity.

Further, we explored whether our approach might be useful in the study of sex-based differences in healthy brain function, with the understanding that this might contribute to the discussion of how and why men and women differ in their expression of mental illness both in prevalence and type. When compared to various existing Q maximization based formulations applied to the same two data sets, PACE yielded results that are both consistent with existing methods yet more stable and reproducible than alternative methods. Moreover, as a result of its superior reproducibility (and thus robustness), PACE was able to detect novel subtle sex differences in resting-state connectivity that were not previously reported with Q-based methods (4). These differences are conceptualized to be the end product of sexual development through hormones, socialization and specialization. Given this and our understanding that brain development may extend into adulthood, in this study we further examined sex differences in resting-state connectome as a function of age (15, 16).

In this study, we comprehensively explored how these differences relate to parcellation resolution as it regards to the validity of these differences. Further, as secondary analyses we investigated how PACE-derived modularity during the resting state may relate to self-reports of common psychopathology traits in relation to mood and anxiety in terms of the sexes. Last, we then proposed a more nuanced framework of conceptualizing the resting state human connectome, termed the basal configuration framework, that generalizes and broadens the narrowly defined and perhaps more restrictive concept of default mode network; this new framework would allow us to better capture the complex dynamic inter-relationship between different brain regions at rest that is further modulated by both age and sex.

## Methodology

### Data

The data we used in this study is 811 subjects' resting state fMRI connectome data from the Human Connectome Project (released in December 2015, named as HCP900 Parcellation+Timeseries+Netmats, https://db.humanconnectome.org/data/projects/HCP_900). Three different spatial dimensions of brain networks are explored: 100 × 100, 200 × 200, and 300 × 300, all derived using independent component analysis or ICA. For study design, recruitment, and enrollment as well as human subject consent and protection, please refer to (3). For details of the dataset and the procedure for connectome construction, please refer to HCP's official website and respective references (3, 17, 18). The study subjects' demographics are shown in Table 1.

**Table 1:** Participant demographics.

**Age**	**Male (Age in years)**	**Female (Age in years)**	**ASR Depressive Problems Raw score Mean ±std (Range)**	**ASR Anxiety Problems Raw score Mean ±std (Range)**	**ASR Inattention Problems Raw score mean ±std (Range)**	**ASR Hyperactivity Problems Raw score Mean ±std (Range)**
22–25	*n* = 106 (23.45 ± 1.08)	*n* = 70 (23.66 ± 1.11)	4.32 ± 3.60 (0–19)	4.05 ± 2.62 (0~11)	3.54 ± 2.44 (0~11)	2.80 ± 2.17 (0~10)
26–30	*n* = 152 (27.91 ± 1.37)	*n* = 197 (28.09 ± 1.48)	4.28 ± 3.74 (0–22)	3.79 ± 2.77 (0–14)	3.03 ± 2.27 (0–14)	2.54 ± 2.04 (0–11)
31–35	*n* = 106 (32.44 ± 1.24)	*n* = 180 (32.86 ± 1.37)	3.62 ± 2.86 (0–15)	3.61 ± 2.43 (0–12)	2.93 ± 2.32 (0–11)	2.24 ± 1.90 (0–8)

### Community estimation

In this study, we adopted the probability associated community estimation (PACE) (4) framework to extract the hierarchical modularity of the resting-state functional connectome (FC). Mathematically represented as an undirected graph *FC*(*V, E*), where *V* is a set of nodes (e.g., ROIs) and *E* the set of edges and given a collection of functional connectomes *S* on *V*, for each edge *e*_*i, j*_ in *E* PACE considers the probability pair: (1) ${P^{+}}_{i,j}$: the probability of observing a co-activating relationship between node *i* and node *j* in *S* (i.e., the tendency that the two regions are active at the same time), and (2) ${P^{-}}_{i,j}$, the probability of observing an anti-activating relationship between node *i* and node *j* in *S*. To estimate this probability pair for each edge, we simply use the ratio between the number of connectomes in *S* having a positive (or negative) correlation values and the total number of connectomes in *S*. Naturally, the *P*^−^- *P*^+^ pair satisfies the following relationship:

$$\begin{array}{l} {P^{-}}_{i,j}+\;{P^{+}}_{i,j}=1,\;\forall \left(i,j\right),\;i\neq j \end{array}$$

Then, given C^1^, C^2^,…, C^N^ that collectively form *an N-way partition* of *V*, PACE denotes the mean intra-community edge positivity or negativity $\bar{P^{\pm }\left(C^{n}\right)}$ for the *n*-th community C^n^ as:

$$\bar{P^{\pm }\left(C^{n}\right)}=\frac{\sum_{i,j\in C^{n},\;i<j}P^{\pm }{\;}_{i,j}\;\;}{\left|C^{n}\right|\left(\left|C^{n}\right|-1\right)/2}$$

Here |*C*^*n*^| denotes the number of nodes in *C*^*n*^. Similarly, the mean inter-community edge positivity and negativity (between communities *C*^*n*^ *and C*^*m*^) are defined as:

$$\bar{P^{\pm }\left(C^{n},\;C^{m}\right)}=\bar{P^{\pm }\left(C^{m},\;C^{n}\right)}=\frac{\sum_{i,j\in C^{n},\;j\in C^{m}}P^{\pm }{\;}_{i,j}\;}{\left|C^{n}\right||C^{m}|}$$

The PACE-modularity is then the partition of *V*, *C*^1^ ∪ *C*^2^ ∪ … ∪ *C*^*N*^ = *V*, (*C*^*i*^ ∩ *C*^*j*^ = ∅ *for all i* ≠ *j*) that maximizes the following benefit function Ψ that computes the difference between mean inter-community and mean intra-community edge negativity (note due to the elementary relationship between *P*^−^ and *P*^+^, PACE can be equivalently thought of as maximizing the difference between the mean intra-community and the mean inter-community edge positivity):

$$\begin{array}{l} \Psi =\underset{C^{1}\cup C^{2}\cup \ldots \cup C^{N}=V,C^{i}\cap C^{j}=\emptyset for\;alli\neq j}{\text{argmax}}\left\{\frac{\sum_{1\leq n<m\leq N}\bar{P^{-}\left(C^{n},\;C^{m}\right)}}{N\left(N-1\right)/2}-\;\frac{\sum_{1\leq n\leq N}\bar{P^{-}\left(C^{n}\right)}}{N}\right\}\\ \;=\underset{C^{1}\cup C^{2}\cup \ldots \cup C^{N}=V,C^{i}\cap C^{j}=\emptyset for\;alli\neq j}{\text{argmax}}\left\{\frac{\sum_{1\leq n\leq N}\bar{P^{+}\left(C^{n}\right)}}{N}-\frac{\sum_{1\leq n<m\leq N}\bar{P^{+}\left(C^{n},\;C^{m}\right)}}{N(N-1)/2}\right\} \end{array}$$

### Constructing the pace null model and testing the statistical significance of each bifurcation and between sexes

**Figure 1:**
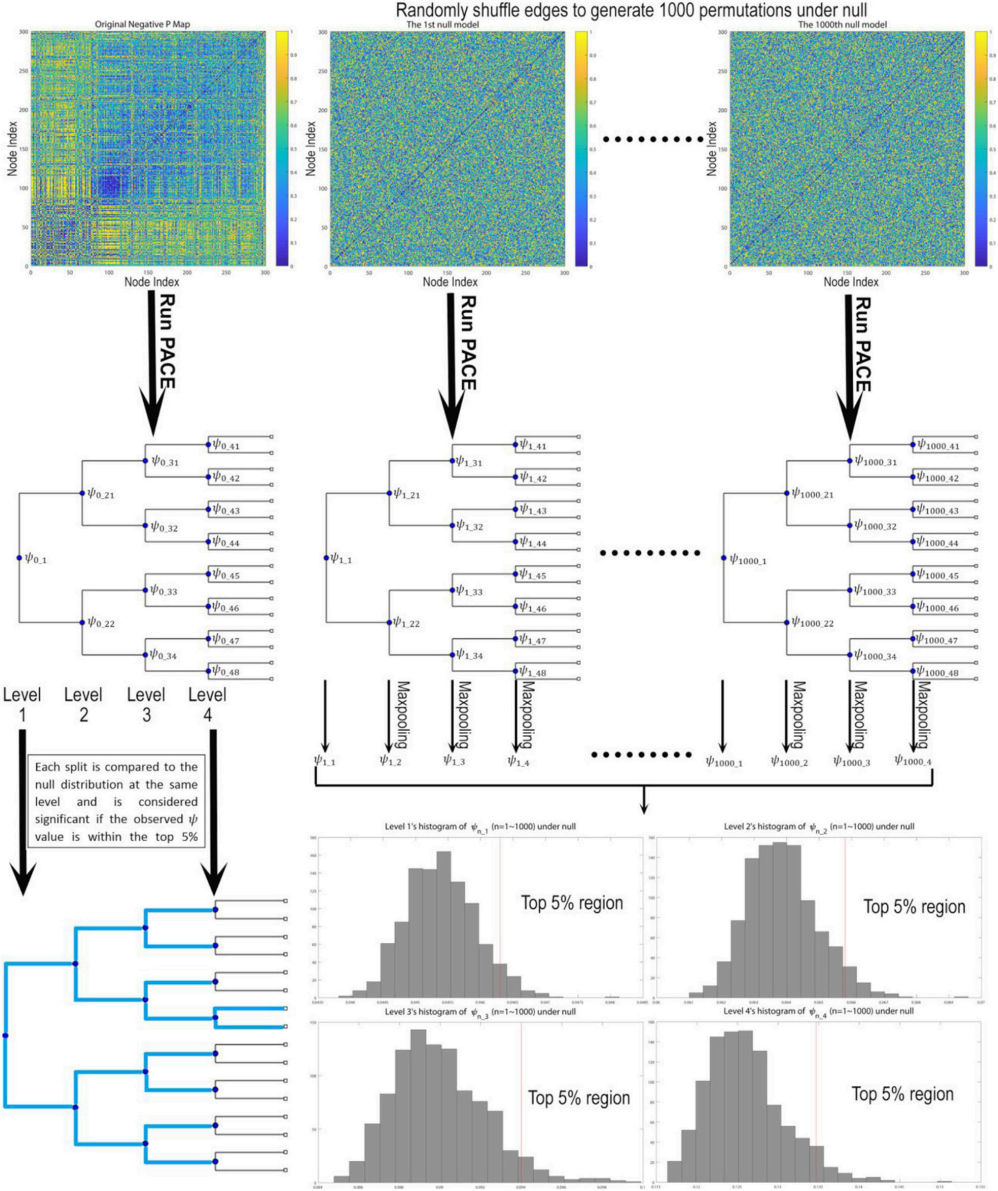
Testing the statistical significance of each bifurcation for the PACE hierarchical modularity tree. First, we derive the community structure from the observed data by maximizing Ψ, defined as the difference between mean inter-community edge negativity and mean intra-community edge negativity. Then under null we assume edge interchangeability, thus allowing us to sample the null distribution of Ψ by randomly shuffling edges to create 1,000 permutations. For each permutation, we re-run PACE to obtain bifurcation trees and record the corresponding Ψ values (and conduct max-pooling for level 2 and up), yielding the null distribution of ψ for each level. Then the bifurcation of the original data at each split will be treated as significant only when the ψ achieved by the original data lies within top 5% with respect to the null distribution.

In our current implementation, a top-down hierarchically bifurcating tree is constructed. For each branch at a specific hierarchical level PACE further splits that branch into 2 subsequent groups by maximizing Ψ with respect to that level using simulated annealing (19, 20). Then, a nonparametric procedure is used to determine the level of statistical significance for such a split. Note that by stopping a branch from further splitting when there is no evidence in support of this bifurcation, PACE can in theory yield any number of communities (i.e., not restricted to powers of 2). This nonparametric procedure leverages the interchangeability of edge positivity/negativity between any two edges under the null distribution given the observed data (since under null there exists no connectome modularity and thus two edges can be randomly selected and exchanged). Thus, assuming there are no modular patterns of co-/anti- activation we could sample the distribution of the PACE benefit function Ψ by first randomly choosing two edges and exchanging their edge negativity/positivity probability pairs (randomization is iterated over the entire connectome) followed by re-computing the PACE trees by maximizing Ψ with reshuffled edges. Then, this entire process is repeated for 1,000 times, yielding 1,000 samples of Ψ under null. Last, to determine the significance of each split, the actual Ψ achieved by the original data is compared to the 1,000 sampled Ψ values under null at the same PACE level; if the former lies within the top 5% of the latter, such a split is determined to be significant (*P* < 0.05) (Figure 1).

To test if PACE modularity differs between sexes, we employed a similar permutation testing approach. Specifically, by permuting with respect to sex (under null the sex of a person is interchangeable) we could recalculate the probability pairs: ${P^{-}}_{i,j},\;{P^{+}}_{i,j}$ at each edge specific to each sex, thus allowing us to generate samples of male/female hierarchical modularity under the null. The difference between sexes is then quantified using the normalized mutual information (NMI) computed between the two sex-specific PACE modular structures and the actual realized sex difference is compared to the resampled differences, thus yielding a p value indicating the level of statistical significance.

### Clinical correlates of connectome modularity: relate systems-level pace modular structure to subject-level characteristics

Firstly, in order to determine the statistical significance of the observed differences between sexes, a 10,000-permutation testing was performed within each age bracket, by first randomly reassigning each subject's sex and then re-computing PACE in order to control for multiple comparisons using the procedure described in Section Constructing the PACE Null Model and Testing the Statistical Significance of Each Bifurcation and Between Sexes.

Then, as part of the HCP data release for behavioral data, we downloaded and used, under the category of Psychiatric and Life Function^1^, the already de-identified Achenbach Adult Self-Report, Syndrome Scales and DSM-Oriented Scale. Here, the Achenbach Adult Self-Report for Ages 18–59 (21) was administered to obtain a broad variety of self-report psychiatric domains. Specifically, the 123 items from Section VIII were used to generate the ASR Syndrome Scales and the ASR DSM-Oriented Scales, and in this study, we primarily focused on Adult Self-Report (ASR) DSM Depressive, Anxiety, Inattention, and Hyperactivity Problems scores (Table 1) and tested if there are sex differences after controlling for age. In particular, using a general linear model incorporating an intercept, main effects, as well as a sex-age interaction term, the statistical significance of a sex effect is tested by centering age within the age range 22–35 across all subjects, for all ASR scores.

Separately, for each of the three resolutions available for HCP (100-ROI, 200-ROI, 300-ROI) we applied PACE to extract modularity (separately for each sex as well as for the combined total sample) and determined the optimal level of bifurcation using the null-model procedure introduced above, thus at the most global level (i.e., 1st level of PACE) yielding two modules. Operationally, as one of the two modules includes regions traditionally considered DMN we will use the term PACE-derived task negative network (TNN) in the remainder of the paper while the other module will be denoted the PACE-derived task positive network (TPN). To summarize the overall activity of TNN and TPN, for each individual we computed the average correlation value within TNN (avg-TNN) and TPN (avg-TPN).

As secondary analyses and to validate our PACE-derived 1st level modules, we explored behavioral correlates of TPN and TNN, we conducted partial correlations, controlling for age, between avg-TNN and avg-TPN and each of the four ASR DSM Problems scores. Previous studies have demonstrated cognitive and affective correlates of TPN and TNN activity associated with the same domains in the ASR DSM scales (22–25).

## Results

### ASR DSM problems scores

For ASR DSM Inattention and Hyperactivity Problems scores men are significantly higher (assessed at mean age of 28.75 years: for Inattention Problems men higher than women by 0.36 points, standard error *SE* = 0.17, *p* = 0.031; for Hyperactivity Problems men higher than women by 0.33 points, *SE* = 0.026, *p* = 0.0023), while women's self-reported ASR Anxiety Problems scores are significantly higher (assessed at mean age of 28.75 years: women higher than men by 0.83 points, *SE* = 0.19, *p* = 8.5e-06). In addition, overall there are age effects for both Anxiety and Hyperactivity Problems scores (lower scores as age progresses: for Anxiety Problems beta = −0.107, *SE* = 0.034, *p* = 0.0017; for Hyperactivity Problems beta = −0.080, *SE* = 0.026, *p* = 0.0023) but not for Inattention Problems score.

For ASR DSM Depressive Problems, there is a significant age effect (beta = −0.137, *SE* = 0.045, *p* = 0.0026) but not a significant sex difference.

### Pace modularity results

**Figure 2:**
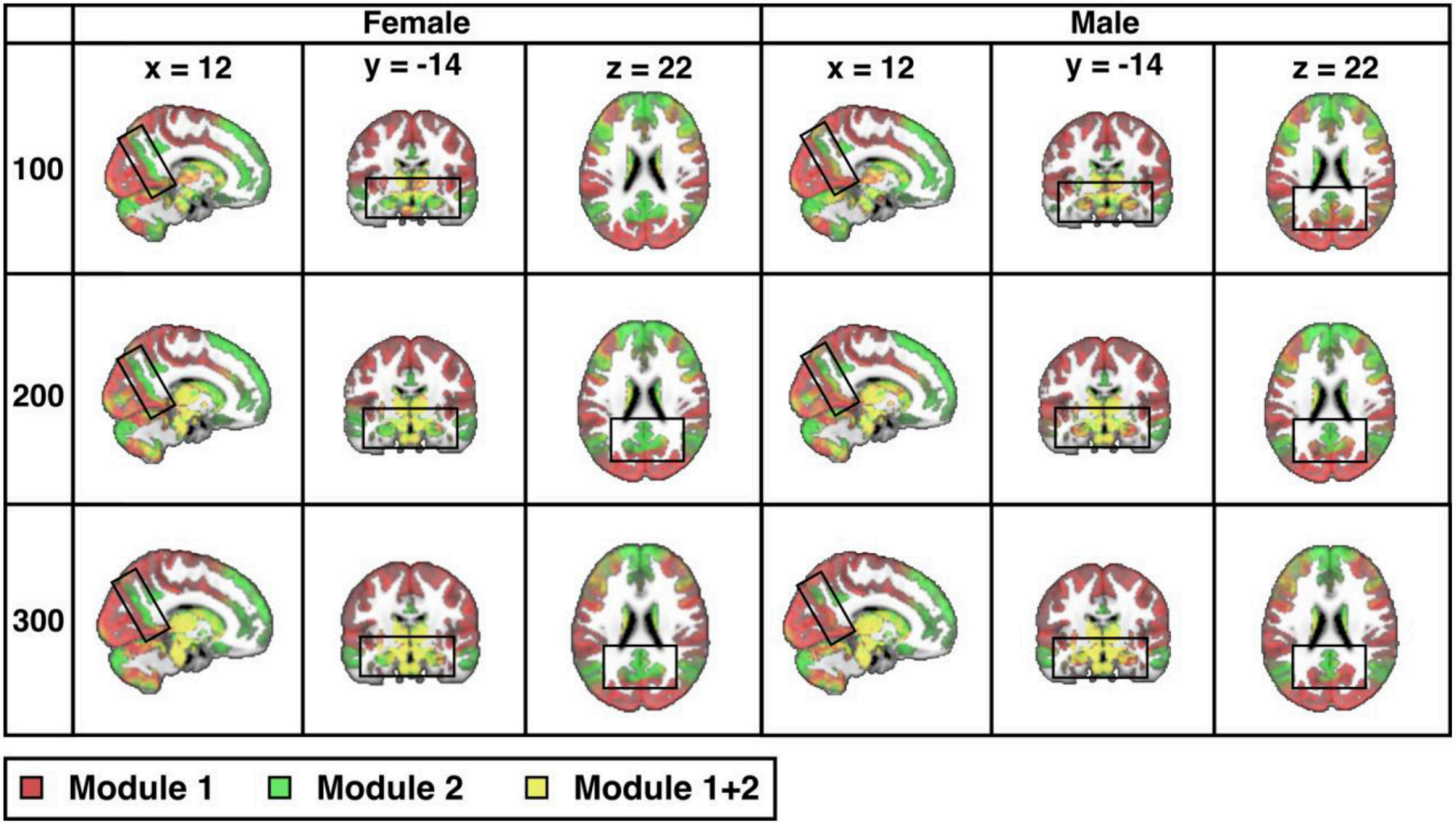
Sex differences in resting-state modularity revealed using the probability associated community estimation (PACE). Slices obtained from 100-ROI, 200-ROI, and 300-ROI resolutions using the Human Connectome Project (HCP) data. At PACE Level 1, two brain modules are extracted, here shown as the red community (corresponding to task positive network or TPN) and the green community (corresponding to task negative community or TNN). Mixing of communities is shown in the overlay in yellow (since HCP parcellation is ICA-based, components may overlap thus resulting in the mixing of TPN and TNN). As expected, with increasing spatial resolution (from 100 to 300 ROIs), sex differences also become more significant (for both 200-ROI and 300-ROI resolution *p* < 0.0001; significant after Bonferroni correction with a p value cut-off 0.05/3). Differences between the sexes (boxed) include precuneus, hippocampus, and amygdala. MNI coordinates: *x* = 12, *y* = −14, *z* = 22.

Across the entire sample, PACE-derived modularity at the most global level (yielding two modules operationally defined as the TPN, in red, and the TNN, in green) is shown in Figure 2 for each sex and each of 3 parcellation resolutions (100-, 200-, and 300- ROIs). As expected, sex differences are visually more easily appreciated for the higher resolutions (*P*-value of sex-differences = 0.0001 for 100 ROIs, and < 0.0001 for both 200 and 300 ROIs).

**Figure 3:**
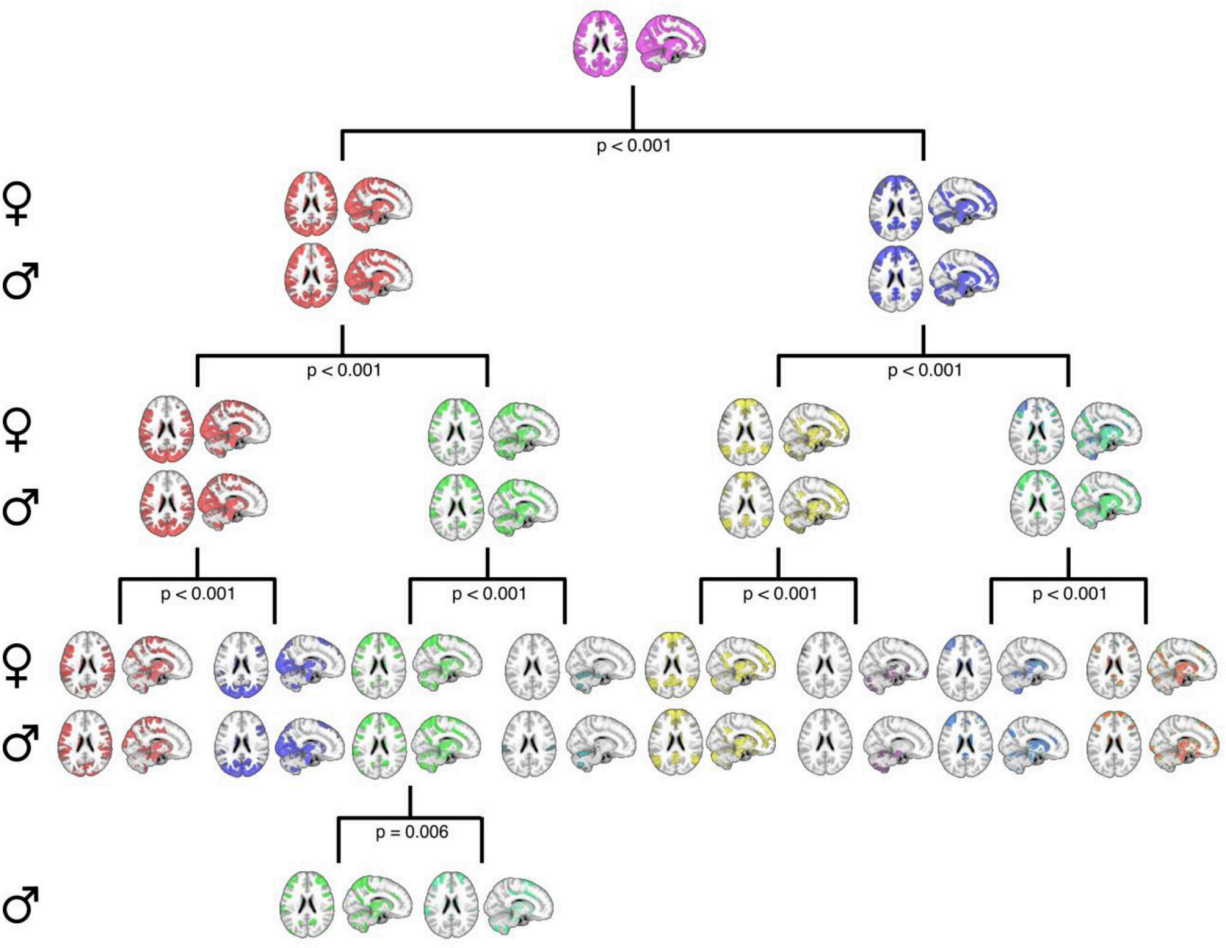
A graphical representation of the PACE-derived male and female hierarchical modular structure, optimally determined using the proposed null-model procedure. Female communities are displayed in the top row of each level with their male counterparts displayed on the bottom row. Axial and sagittal slices are shown for each community. Note that while female community bifurcation was no longer significant after PACE Level 3 (eight total communities), a further significant bifurcation was observed in males (third community from left at PACE level 3; *p* = 0.006), yielding nine total communities. MNI coordinates: *x* = 12, *z* = 22.

Second, Figure 3 details the corresponding complete hierarchical modularity for men and women across all three age groups, visualized as bifurcation trees, after applying our null-distribution procedure (see Section Constructing the PACE Null Model and Testing the Statistical Significance of Each Bifurcation and Between Sexes). Interestingly, we identified an additional split in men during this procedure (resulting in 8 modules for women and 9 for men).

### Pace modularity as a function of age

**Figure 4:**
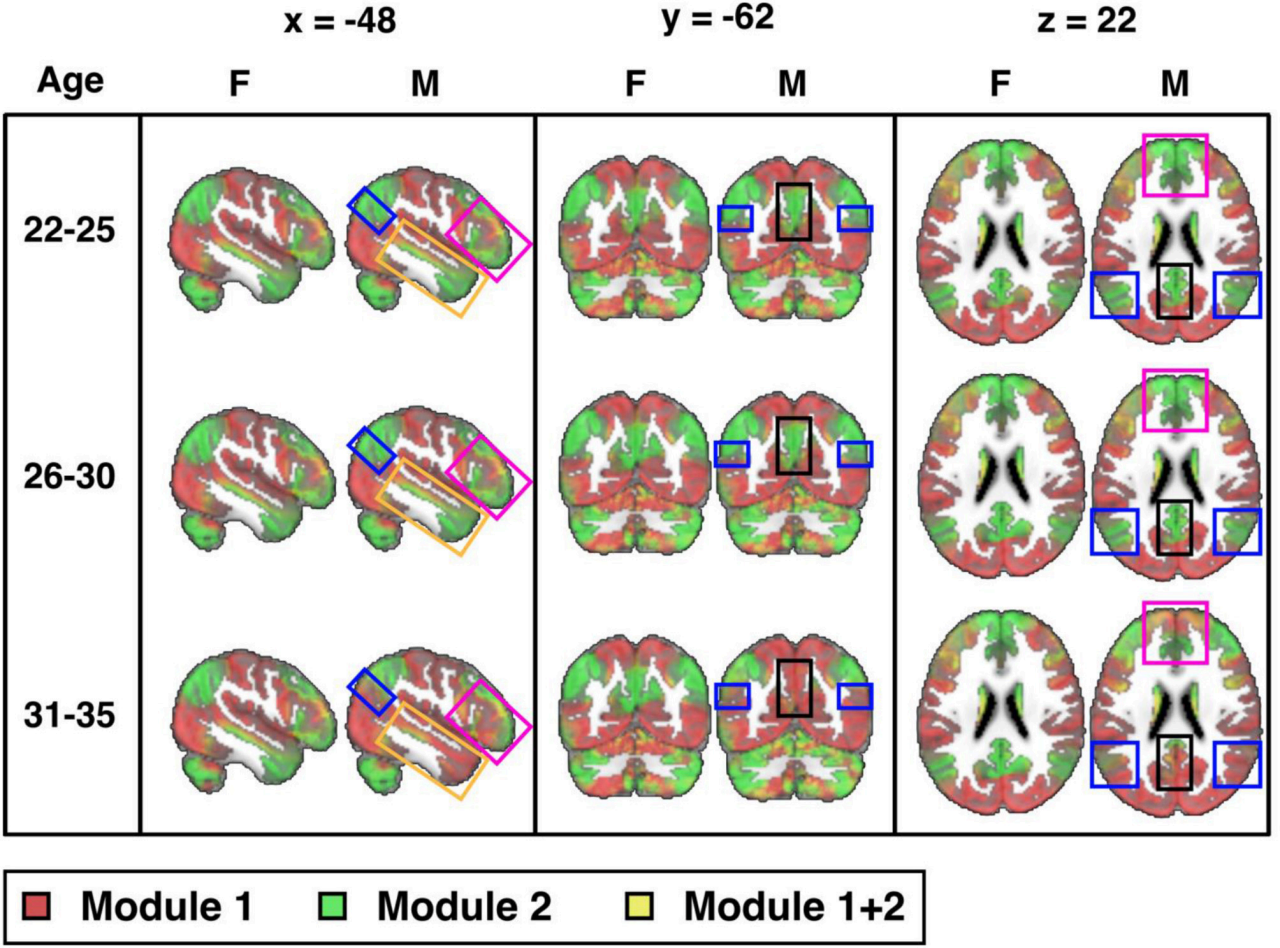
Sex differences across three age groups (visualized at PACE Level 1): 22-25 (M: 106, F: 70), 26-30 (M: 152, F: 197), and 31-35 (M: 106, F: 180) years-old (cf. Table 1) from the 300-ROI HCP data. Sex differences were not statistically significant in the 22-25 years old group, but reached statistical significance in the 26-30 years old group (p = 0.003; significant after Bonferroni correction at a p value cut-off of 0.05/3) and is also statistically significant in the 31-35 group (p < 0.001). Areas of significant differences are in brain regions where transitions occur from TNN (green) to TPN (red) in men but not in women (boxed areas: black: precuneus; blue: inferior parietal lobule; magenta: prefrontal cortex; orange: middle temporal gyrus). F = female; M = male. MNI coordinates: x = −48, y = −62, z = 22. Note, instead of indicating voxel-wise differences between sexes, the highlighted areas localize where modularity as a network property differs significantly.

Next, we explore connectome modularity as a function of age, with results shown in Figure 4 where we visualize the sex-specific modularity in each of three age groups (22–25, 26–30, and 31–35). Although sex-differences did not reach statistical significance in the 22–25 years old age group, visual differences were noted in several areas. Sex-differences then reached statistical significance in the 26–30 years old age group (*p* = 0.003) and even more significant in the 31–35 age group (*p* < 0.001). Interestingly, female modularity remains largely stable across the three age groups (a head-to-head comparison between the 22 and 25 y/o group and the 31–35 y/o group was indeed statistically not statistically significant), whereas males exhibit different patterns across age groups, particularly from the 26–30 to 31–35 age groups. Also, the areas of significant differences in men are in brain regions where “transitions” occur, as they age, gradually from more probabilistically task-negative (green) into task-positive (red) (including the precuneus, the inferior parietal lobule, the prefrontal cortex, the hippocampus, the amygdala, and the middle temporal gyrus; see Supplemental Video).

### Secondary analyses: correlation between pace modularity and ASR DSM problems scores

Secondary *post-hoc* partial correlation analyses (controlling for age) confirmed that avg-TPN is negatively correlated with ASR Anxiety and Inattention Problems scores in men, but not in women; all other correlations are statistically non-significant (*r* = −0.131 and *p* = 0.01 for Anxiety Problems, *r* = −0.127 and *p* = 0.015 for Inattention Problems; *p*-values uncorrected).

**Figure 5:**
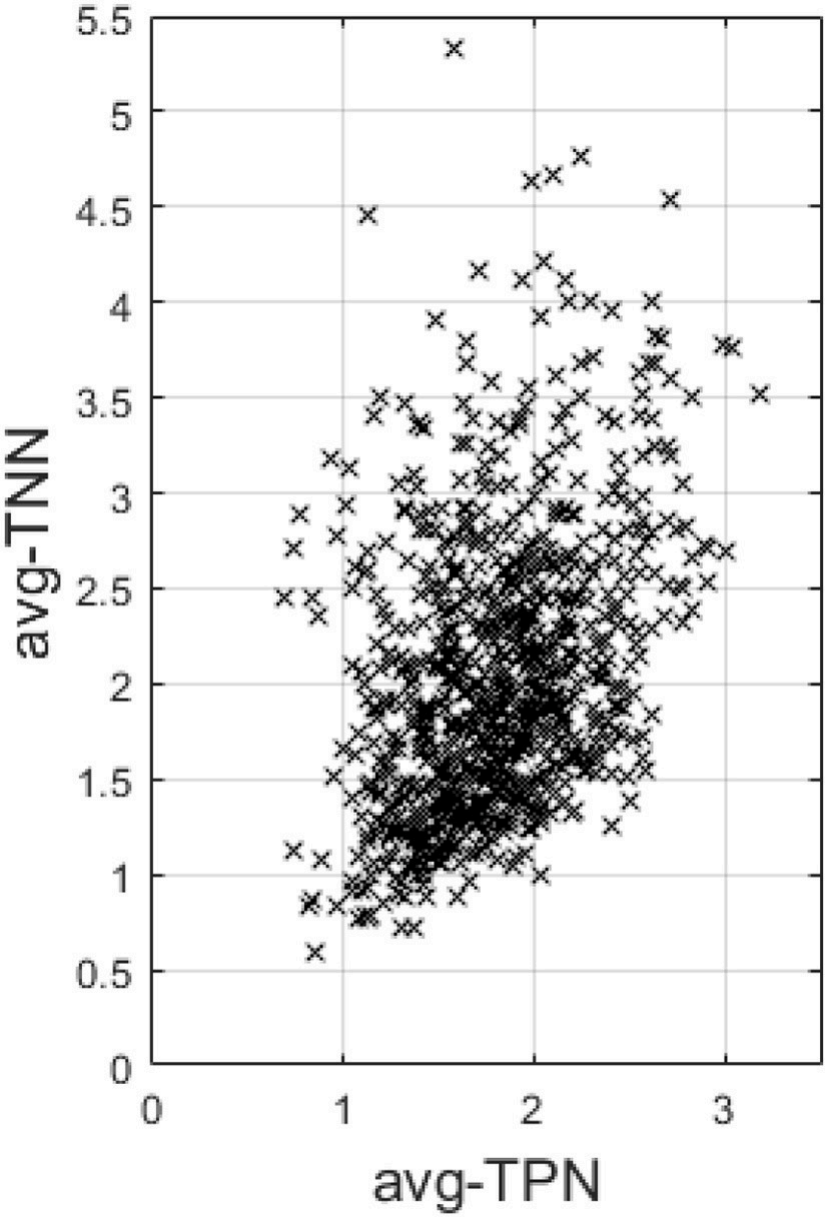
This figure plots the average Fisher's z-transformed correlation within TNN (avg-TNN) against that computed within TPN (avg-TPN) across the entire sample. There is a highly significant positive correlation (r = 0.37 and p = 1.4e-28) between the two, suggesting a synergistic relationship between them than antagonistic.

Last, across the entire sample, avg-TPN also positively correlates with avg-TNN (*r* = 0.37 and *p* = 1.4e−28, Figure 5).

## Discussion

In this study, using the F1000 and the HCP dataset we comprehensively examined resting-state connectome sex differences over the course of early adulthood, in particular how they are modulated by age as well as their clinical correlates. Our findings lead to a more nuanced picture elucidating: (1) how fMRI-derived brain connectivity exhibits both sex-specific configuration and age-dependent dynamic re-configuration, the latter primarily in men, from early twenties to mid-thirties, and (2) how this sex-by-age configuration during the resting state relates to self-reports of common psychopathology traits in otherwise healthy subjects.

Although sex-differences were not identified at a level of statistical significance in the 22–25 years old age group, a trend difference is observed in several areas in the brain. Specifically, in areas either designated as or functionally closely coupled with DMN we identified a gradually shifting connectome configuration over time in men (vs. women), away from probabilistically more “task negative” and toward probabilistically more “task positive.” In this sense, our probabilistic framework frees up a brain region from having to be hard-parsed (e.g., DMN vs. non-DMN), thus allowing functional brain units (i.e., modules) to work synergistically at times, and to act nearly-decomposable at other times. Of interest, at the same time that basal configuration differences are identified with increasing statistical significance with age, the differences also show increasing statistical significance within individual age groups with increasing image resolution.

What might contribute to the sex differences observed in the present study? Some studies have shown that hormones like estrogens and progesterone might modulate the resting-state functional connectivity within the DMN (26, 27). Further in support of the argument that hormones play a role in connectivity differences, oral contraceptives taken by women are known to impact functional activation to emotional stimuli and resting state functional connectivity (26, 28, 29), while women on oral contraceptives may have smaller hippocampal volume (30).

The results of our study indicate that the differences between men and women do not appear statistically significant until we reach the 26–30 years old age range and the trend appears to increase into the 31–35 years old age group. This is well past the age of puberty and so the first question to arise is why do these differences gain statistical significance at an age range substantially older than the onset of puberty?

One likely hypothesis is that men move to develop sexual dimorphism in function later in adulthood, perhaps through later development of white matter connections than women. Along these lines, one recent study suggested that the 20–25 years old age range may be a point of inversion of earlier sex differences in cortical thickness and folding differences, however this study was limited in that they did not have a cohort extending into the 30s to fully probe this potential interaction between age and sex (31).

Given these sex-based age-dependent differences in otherwise healthy people, a natural further course of inquiry is to ask how these differences might relate to the known differences in the prevalence and symptom expression of mental illness between the sexes. Among clear differences between men and women in terms of mental health is that women are much more likely than men to experience episodes of depression (32–35) Examples of frequently investigated factors are differences in: hormonal development, societal roles, communication patterns and ways of coping with stressors. However, the lack of definitive conclusions to date suggests that additional and unrecognized factors might be at play (34). As such, it may be useful to consider whether sex-based differential brain network connectivity might be one of these unrecognized factors. Clearly it is a complex picture and it is important to note again that exposure to sex hormones is already known to affect brain network connectivity. One example of this is discussed by Ottowitz et al., that addition of estrogen to post-menopausal women is shown to increase connectivity between the hippocampus and the prefrontal cortex, which as part of the fronto-limbic circuitry known to play important roles in depression, (36) while Maki et al. provide another example in which they find that perimenopausal women exposed earlier to hormone replacement therapy perform better in various cognitive tasks than those who are exposed after a longer time untreated (37).

One area in which differential brain network connectivity possibly contributes to sex-based differences in depression is rumination. Rumination is the repetitive thinking and focus on negative mood states (38). It is also a form of self-referential processing of information (39). Areas identified as playing a role in self-referential processing have included: medial prefrontal cortex, anterior and posterior cingulate cortex, insula, temporal pole, hippocampus, and amygdala (32, 39–47); note that in our current study we identified sex-based differences in areas highly overlapping with these.

Another major difference recognized in our study is in regard to self-reported inattention, hyperactivity, and anxiety problems scores. While depression problems scores are similar between the sexes, men have higher self-reported inattention and hyperactivity scores than women (women by contrast have higher overall anxiety problems scores). Further, correlation analyses revealed that overall average-TPN is positively correlated with average-TNN, and in the male group the former further inversely correlated with attention problems and anxiety problems scores.

Last, note that our sex-difference findings in self-reports are generally in line with Gur and Gur's paper in which they found that men have earlier declines in frontotemporal areas associated with attention, inhibition and memory (48). Indeed, men tend to express symptoms of depression differently than women in terms of acting-out behaviors such as substance abuse, restlessness and suicide (34, 49). While this difference in expression of depressive symptoms is not fully understood, it is possible that the tendency toward higher degrees of inattention and hyperactivity in otherwise healthy men at least partially explains why men tend to exhibit more externalizing behaviors when depressed. This finding is also interesting to consider in the context of Ingalhalikar et al.'s findings regarding sex-based connectivity differences in adolescents in which the development of greater inter-hemispheric connectivity in females leads to better performance in attention tasks (50).

There are a few limitations in our current study. First, while PACE is a rigorous framework to model negative correlations and to pinpoint modular differences in a hierarchical fashion, it is only able to localize on a modular level where groups differ (but not on a voxel level). Second, the fact that we did not find any sex difference in the youngest age range may be simply due to a smaller sample size. Similarly, the fact that we only found basal configuration correlations with self-reports in the male group merits more discussion. While this can be due to a lack of power in the female group, we did find modestly robust correlations in the male group. Alternatively, as recent lines of evidence suggesting that in women cognition is a function of the menstrual cycle due to differential effects of estrogen and progesterone (51), another plausible explanation is that in women the basal configuration and self-reports such as ASR scores are also dependent on the menstrual cycle. If so, the lack of correlations in the female group is merely a consequence of the fact that the functional imaging data and the self-reports were obtained on different days and/or were differentially dependent on phases of the menstrual cycle. Future studies can use a repeated measures framework to estimate the relationship between change in the basal configuration in relation to these self-reports and other potential factors that may develop later or differently in males.

## Conclusion

In sum, this study contributes to a growing literature on the limitations of our current conceptualization of resting-state networks. Our research supports the argument that conceptualizing the default mode network as being the network active at rest, thus driving resting-state brain dynamics, while the activation of other networks is responsible for taking over during active tasks is a concept that requires continued consideration. In time, it is possible that a more nuanced model of functioning will be identified as the leading theory on brain connectivity. In this fashion, we argue that the current perspective is an oversimplification of the actual complexity of brain connectivity, as on a subject-level with increasing default mode network activity the functional activity in other networks (generally considered to be “task positive” networks) increases as well. Taken in conjunction with previously published studies in which increasing connectivity is an important factor in overall functionality, (18) this study lays the foundation of a more nuanced framework for better conceptualizing the resting state. To this end, our results supported that: (1) the basal configuration exhibits distinct sex-specific dynamics by mid 30s, (2) the basal configuration diverges during early adulthood between the sexes, in that there is globally an age-modulated reconfiguration primarily in men but not women, (3) the basal configuration correlates with self-reported measures of personality traits, at least in men, (4) whereas in women the basal configuration is further conjectured to likely exhibit a strong dependence on the menstrual cycle.

## Author contributions

All authors wrote the paper. LZ, ZM, MX, AL designed the study and conducted the calculations. AF, PM, MM, OA, and SL provided the discussion on the results.

### Conflict of interest statement

The authors declare that the research was conducted in the absence of any commercial or financial relationships that could be construed as a potential conflict of interest.

## Footnotes

^1^https://wiki.humanconnectome.org/display/PublicData/HCP+Data+Dictionary+Public-+Updated+for+the+1200+Subject+Release#HCPDataDictionaryPublic-Updatedforthe1200SubjectRelease-RestrictedInstrument:LifeFunction(AchenbachAdultSelf-Report,SyndromeScalesandDSM-OrientedScale).

## Supplementary material

Supplemental Video Sliding window of age-by-sex differences. Images were constructed with MRIcron using PACE Level 1 (two communities) applied to the 300-ROI HCP900 data using ten age windows for each sex (22–25, 23–26, 24–27, 25–28, 26–29, 27–30, 28–31, 29–32, 30–33, 31–34, 32–35). Each image duration is 2 s, with 1 s cross-dissolve transition between images. Age group is indicated via a text label at their respective time stamps. MNI coordinates of the images used were x = 52, y = 57, z = 48.
